# Comparison of Transmittance and Reflectance Pulse Oximetry in Anesthetized Dogs

**DOI:** 10.3389/fvets.2021.643966

**Published:** 2021-04-30

**Authors:** Jan Nixdorff, Yury Zablotski, Katrin Hartmann, Rene Dörfelt

**Affiliations:** Clinic of Small Animal Medicine, Centre for Clinical Veterinary Medicine, Ludwig-Maximilians-University (LMU) Munich, Munich, Germany

**Keywords:** oxygen saturation, hypoxia, tail, tibia tongue, canine

## Abstract

**Objectives:** The tongue is the standard site for placement of a pulse oximeter probe but is difficult to access during certain procedures such as dental and ophthalmic procedures and computerized tomography of the head. The aim of this study was to evaluate the performance of a new-generation reflectance pulse oximeter using the tail and tibia as sites for probe attachment.

**Materials and Methods:** A total of 100 client-owned dogs that underwent anesthesia for various reasons were premedicated with butorphanol (*n* = 50; 0.2 mg/kg; group BUT) or butorphanol and dexmedetomidine (*n* = 50; 5 μg/kg; group DEX), administered intravenously. Anesthesia was induced with propofol and maintained with sevoflurane. A transmittance pulse oximeter probe was placed on the tongue and served as the reference standard. A reflectance probe was randomly placed on the tail base or the proximal tibia, and the position changed after testing. Signals from three consecutive measurements were obtained at each position. Failure was defined as “no signal,” “low signal,” or a pulse difference >10/min compared with the ECG heart rate. Data were analyzed using chi-square test, Wilcoxon matched-pair signed-rank test, and Bland-Altman analysis. *P* < 0.05 was considered significant.

**Results:** In both groups (BUT and DEX), failure rate was higher when the tibia and tail were used as probe sites compared with the tongue. In both groups, the failure rate was higher for the tibia than for the tail. Dexmedetomidine-induced vasoconstriction increased failure rate at all probe positions.

**Clinical Significance:** The tail base, but not the tibia, is an acceptable position for reflectance pulse oximeter probes in dogs. The tongue remains the probe site of choice, if accessible.

## Introduction

Pulse oximetry is considered a standard tool for monitoring veterinary patients during anesthesia. It was shown to detect ~40–82% of peri-anesthetic incidents (1) and to significantly reduce anesthesia-related mortality in cats (2).

In veterinary medicine, transmittance pulse oximetry is the most commonly used method and involves emission of red and infrared light, which passes through the tissues from one side of the probe to a receiver on the opposite side. A computer program analyzes the amount of light absorbed by the tissues to calculate the pulse rate and oxygen saturation (3).

The standard site for probe placement using transmittance pulse oximetry is the tongue in dogs. However, procedures such as dental or ophthalmologic surgery and diagnostic imaging of the head make attachment of a probe to the tongue difficult or impossible. A plausible solution is the use of reflectance pulse oximetry, which is commonly applied in human medicine at the chest wall, the foot sole, and the forehead as probe placement sites (3–7). In reflectance pulse oximetry, the probe emits infrared and red light, which passes through the tissues, is reflected by the underlying bone, and detected at the level of the light-emitting electrodes. Reflectance pulse oximetry has been used empirically in dogs with the medial aspect of the proximal tibia and the ventral part of the tail base used as probe placement sites.

The aim of this study was to evaluate the performance of a reflectance pulse oximeter probe placed on the medial aspect of the proximal tibia and the ventral part of the tail base in anesthetized dogs that had or had not been premedicated with a vasoactive drug. A transmittance pulse oximeter probe placed on the tongue served as the reference standard.

## Materials and Methods

The study protocol was accepted by the ethics committee of the Center of Clinical Veterinary Medicine (number 16-04-10-13).

### Animals

One hundred client-owned dogs that underwent general anesthesia for various diagnostic or therapeutic procedures were included in the study. Dogs were excluded if they weighed <2.5 kg or had respiratory disease, anemia, dyshemoglobinemia, a mean arterial blood pressure <60 mmHg during anesthesia, pathologic arrhythmia, body temperature <37.0°C during anesthesia, received vasoactive drugs before anesthesia, or when anesthesia would have been unduly prolonged because of the study.

The study included 38 intact male, 20 castrated male, 26 intact female, and 16 spayed female dogs. The most common breeds were mixed breed (8), Labrador Retriever (7), and West Highland White Terrier (5) with other breeds represented <5 individuals per breed. The median age was 6.6 years (0.3–15.0 years), and median body weight was 21.7 kg (2.5–53.0 kg).

In all dogs, the medical history was recorded and a standardized preanesthetic examination was carried out to determine the American Society of Anesthesiologists (ASA) physical status.

### Anesthesia

Dogs were prospectively assigned to one of two premedication groups (BUT or DEX) according to their ASA status. The BUT group consisted of 50 dogs that had an ASA status of 3 and were premedicated with butorphanol 0.2 mg/kg, administered intravenously (IV). The DEX group consisted of 50 dogs that had an ASA status of 1 or 2 and were premedicated with dexmedetomidine 5 μg/kg^1^ and butorphanol 0.2 mg/kg (group DEX)^2^, administered IV, to evaluate the effect of vasoconstriction. Anesthesia was induced with propofol (2–6 mg/kg)^3^, administered IV to effect in both groups. Dogs were intubated with a cuffed low pressure–high volume endotracheal tube^4^, and anesthesia was maintained with sevoflurane^5^ in 100% oxygen. The sevoflurane concentration was adjusted according to the depth of anesthesia, which was based on jaw tone, bulbus position, and ocular reflexes. A multiparameter monitor^6^ was used to evaluate end-tidal CO_2_, end-tidal sevoflurane, cardiac activity (ECG), noninvasive blood pressure, and rectal body temperature during anesthesia. All dogs had spontaneous breathing initially, but pressure-controlled mechanical ventilation was started if end-tidal CO_2_ was >45 mmHg with a maximum inspiratory pressure of 8 mbar. Ventilator settings were adjusted to maintain an end-tidal CO_2_ of 35–45 mmHg. The position of the dogs depended on the requirements of the procedure and was recorded.

### Reference Pulse Oximeter

A new-generation pulse oximeter^7^ equipped with a transmittance probe^8^ was chosen as the reference standard. The transmittance probe was placed on the lateral aspect of the tongue.

### Pulse Oximeter With a Reflectance Probe

A second pulse oximeter produced by the same manufacturer and of the same type^7^ was equipped with a reflectance probe^9^. The reflectance probe was placed on the ventro-cranial aspect of the tail or the medio-proximal aspect of the tibia in random order, and then the location was changed after recordings were completed. A 2 × 2 cm area of hair was clipped at the probe placement sites, and the probe was fixed in place using self-adhesive bandage material. Both pulse oximeters used in this study had been tested in a preliminary study and found to have a similar performance.

### Measurements

Parallel measurements were obtained with the transmittance probe placed on the tongue and the reflectance probe placed on either the tail base or the tibia, in random order. The pulse oximeter displays and the multiparameter monitor were video recorded three times if the pulse oximeter signal had stabilized after a 1-min equilibration period. After three videos were recorded, the probe position of the reflectance probe was changed to the alternate position and pulse oximeter signals of both probes were recorded again. Pulse rate, oxygen saturation measured by pulse oximetry (SpO_2_), signal quality (SQ), and perfusion index (PI) values were analyzed from the videos. The heart rate of the dogs was recorded from the ECG. When a large deviation between the ECG heart rate and pulse oximeter pulse rate occurred, the heart rate was confirmed by manual auscultation of the heart.

### Data Analysis

The results were classified as failure of the device when the pulse rate or SpO_2_ were not displayed, the signal intensity was ≤ 2 of 10 signal intensity bars, or the pulse rate differed by >10 beats per minute from the ECG heart rate. Statistical analysis was done using a commercial software^10^. Normality was analyzed by the D'Agostino-Pearson test. Non-parametric, non-normally data were presented as median (m) and range. Differences in age were analyzed by Mann-Whitney *U*-test. Failure rate was analyzed by Chi-square test. Wilcoxon matched pairs signed rank sum test, Mann-Whitney *U*-test, and Bland-Altman analysis were used to compare SpO_2_, pulse rate, and heart rate, signal quality, and perfusion index. A *P* ≤ 0.05 was considered significant.

## Results

The failure rate in groups BUT and DEX was higher when the reflectance oximeter probe was placed on the tibia (BUT and DEX: *P* < 0.0001) and the tail (BUT: *P* = 0.0007; DEX *P* < 0.0001) compared with the transmittance oximeter probe placed on the tongue. In both premedication groups (BUT and DEX), the failure rate was lower when the probe was on the tail than when it was on the tibia (*P* < 0.0001; Table 1).

**Table 1:** Failure rate of 600 pulse oximeter measurements with a transmittance pulse oximeter probe placed on the tongue and a reflectance pulse oximeter probe placed on the tail or tibia in 100 anesthetized dogs.

**Total**			**Transmittance probe tongue**		**Reflectance probe tail**		**Reflectance probe tibia**		***P*****-value**		
	***n***	**Failure rate**	***n***	**Failure rate**	***n***	**Failure rate**	***n***	**Failure rate**	**Tongue vs. tail**	**Tongue vs. tibia**	**Tail vs. tibia**
BUT	600	14.3%	300	3.7%	150	12.0%	150	38.0%	**0.0007**	**<0.0001**	**<0.0001**
DEX	600	27.5%	300	12.5%	150	28.0%	150	57.3%	**<0.0001**	**<0.0001**	**<0.0001**

*n, number of measurements; BUT, premedication butorphanol 0.2 mg/kg IV; DEX, premedication dexmedetomidine 5 μg/kg + butorphanol 0.2 mg/kg IV*.

*Measurement failure was defined as: pulse rate or SpO_2_ not displayed, signal intensity ≤2 of 10 signal intensity bars, or pulse rate differed from ECG heart rate by >10 beats per minute. Bold values mean significant differences*.

Median SpO_2_ values in group BUT where higher when the probe was on the tail (*P* < 0.0001) and tibia (*P* < 0.0001) compared with measurements from the tongue. There was no difference in SpO_2_ between the tail and the tibia probe locations (*P* = 0.2003). Median SpO_2_ in group DEX was higher when the probe was on the tail compared with measurements from the tongue (*P* < 0.0001), not different between the tongue and the tibia sites (*P* = 0.3366), and higher for the tail compared with the tibia (*P* = 0.0046; Table 2). Bias (mean difference) but not 95% limit of agreement (LoA) was lowest for comparisons of tongue and tibia measurements. In group DEX, the lowest BIAS was observed in comparisons of tongue and tail measurements. In group BUT, the lowest 95% LoA was observed in comparisons of tongue and tail measurements (Table 3).

**Table 2:** Analysis of oxygen saturation measured by a transmittance pulse oximeter probe placed on the tongue or by a reflectance pulse oximeter probe positioned on the tail or tibia in anesthetized dogs that had or had not received dexmedetomidine.

	***n***	**SpO_**2**_ m (%) (min-max%)**	**SpO_**2**_ m (%) (min-max%)**	***P*-value**
**Tongue vs. tail**		**Tongue**	**Tail**	
BUT	132	96 (90–100)	100 (89–100)	**<0.0001**
DEX	100	97 (87–100)	99 (67–100)	**<0.0001**
**Tongue vs. tibia**		**Tongue**	**Tibia**	
BUT	92	96 (77–100)	100 (93–100)	**<0.0001**
DEX	69	96.5 (82–100)	98.5 (70–100)	0.3366
**Tail vs. tibia**		**Tail**	**Tibia**	
BUT	85	100 (93–100)	100 (74–100)	0.2003
DEX	49	97 (70–100)	99 (78–100)	**0.0046**

*Values were only included if measurements for both positions were available and analyzed as paired values*.

*n, number of measurements; tongue, transmittance pulse oximeter probe placed on the tongue; tail, reflectance pulse oximeter probe placed on the ventral tail base; tibia, transmittance pulse oximeter probe placed on the medial aspect of the proximal tibia; SpO_2_, oxygen saturation measured by pulse oximeter; BUT, premedication butorphanol 0.2 mg/kg IV; DEX, premedication dexmedetomidine 5 μg/kg + butorphanol 0.2 mg/kg IV; m, median. Bold values mean significant differences*.

**Table 3:** Bland-Altman analysis of the pulse oximetry values displayed by a transmittance probe placed on the tongue and a reflectance probe placed on the base of the tail in anesthetized dogs.

**Tongue vs. tail**	**All**		**BUT**		**DEX**	
	**BIAS**	**95% LoA**	**BIAS**	**95% LoA**	**BIAS**	**95% LoA**
SpO_2_ (%)	−2.2 ± 3.8	−9.7–5.2	−3.1 ± 2.6	−8.3–2.0	1.0 ± 4.7	−8.2–10.2
PR (/min)	−0.1 ± 3.2	−6.1–6.1	−0.3 ± 2.4	−5.0–4.5	0.0 ± 4.0	−7.8–7.8
**Tongue vs. tibia**						
SpO_2_ (%)	−0.6 ± 7.2	−14.8–13.6	−2.0 ± 6.8	−15.5–11.4	1.6 ± 7.3	−12.8–16.0
PR (/min)	−0.2 ± 2.7	−5.2–5.5	−0.1 ± 2.9	−5.7–5.5	0.5 ± 2.4	−4.3–5.3
**Tail vs. tibia**						
SpO_2_ (%)	−2.2 ± 6.9	−11.4–15.7	−0.8 ± 4.5	−8.1–9.8	4.4 ± 9.4	−14.0–22.8
PR (/min)	−3.7 ± 10.8	−24.8–17.5	−3.3 ± 10.3	−23.5–16.8	4.2 ± 11.7	−27.1–18.7

*All, all dogs; BUT, dogs premedicated with butorphanol 0.2 mg/kg IV; DEX, dogs premedicated with dexmedetomidine 5 μg/kg IV and butorphanol 0.2 mg/kg IV; 95% LoA, 95% limits of agreement; SpO_2_, oxygen saturation measured by pulse oximetry; PR, pulse rate measured by pulse oximetry; SQ, signal quality measured by pulse oximetry; PI, perfusion index measured by pulse oximetry*.

Pulse rate differed from heart rate (ECG) only in group BUT with measurements from the transmittance probe on the tongue (*P* = 0.0195; Table 4). Pulse rate did not differ between the tongue and tail sites in both the BUT and DEX groups (*P* = 0.0261; DEX: *P* = 0.0076; Table 5). BIAS for heart rate was lowest for comparisons of measurements from the tongue and tail in group DEX. Lowest 95% LoA was observed for comparisons of measurements from the tongue and tail in group in BUT (Table 3).

**Table 4:** Comparison of pulse rate, measured by a transmittance pulse oximeter probe placed on the tongue or by a reflectance pulse oximeter probe placed on the tail or tibia and heart rate measured by ECG in anesthetized dogs that had or had not received dexmedetomidine.

	***n***	**Pulse rate m (/min) (min-max/min)**	**Heart rate m (/min) (min-max/min)**	***P*-value**
**Tongue vs. ECG**		**Tongue**	**ECG**	
BUT	226	102 (35–157)	102.5 (39–161)	**0.0195**
DEX	100	65.5 (32–106)	65 (32–106)	0.7106
**Tail vs. ECG**		**tail**	**ECG**	
BUT	132	101.5 (36–157)	102 (39–157)	0.4567
DEX	60	71 (33–110)	71 (30–113)	0.1209
**Tibia vs. ECG**		**tibia**	**ECG**	
BUT	92	102.5 (52–149)	104.5 (52–161)	0.4793
DEX	49	75 (37–106)	77 (57–113)	**0.0076**

*Values were only included if measurements for both positions were available and analyzed as paired values. Pulse rate values that differed from the ECG by >10 beats per minute were excluded*.

*n, number of measurements; tongue, transmittance pulse oximeter probe placed on the tongue; tail, reflectance pulse oximeter probe placed on the ventral tail base; tibia, transmittance pulse oximeter probe placed on the medial aspect of the proximal tibia; HR, heart rate; PR, pulse rate; BUT, premedication butorphanol 0.2 mg/kg IV; DEX, premedication dexmedetomidine 5 μg/kg + butorphanol 0.2 mg/kg IV; m, median. Bold values mean significant differences*.

**Table 5:** Analysis of the pulse rate measured by a transmittance pulse oximeter probe placed on the tongue or by a reflectance pulse oximeter probe placed on the tail or tibia in anesthetized dogs that had or had not received dexmedetomidine.

	***n***	**Pulse rate m (/min) (min-max/min)**	**Pulse rate m (/min) (min-max/min)**	***P*-value**
**Tongue vs. tail**		**Tongue**	**Tail**	
BUT	132	102 (35–157)	101.5 (36–157)	0.0962
DEX	100	65.5 (32–106)	65 (32–106)	0.7106
**Tongue vs. tibia**		**Tongue**	**Tibia**	
BUT	92	103 (52–157)	102.5 (52–149)	0.5665
DEX	60	71 (33–110)	71 (30–113)	0.1209
**Tail vs. tibia**		**Tail**	**Tibia**	
BUT	85	102 (47–147)	104 (52–148)	**0.0261**
DEX	49	75 (37–106)	77 (57–113)	**0.0076**

*Values were only included if measurements for both positions were available and analyzed as paired values*.

*n, number of measurements; PR, pulse rate; tongue, transmittance pulse oximeter probe placed on the tongue; tail, reflectance pulse oximeter probe placed on the ventral tail base; tibia, transmittance pulse oximeter probe placed on the medial aspect of the proximal tibia; BUT, premedication butorphanol 0.2 mg/kg IV; DEX, premedication dexmedetomidine 5 μg/kg + butorphanol 0.2 mg/kg IV; m, median. Bold values mean significant differences*.

In group BUT, signal quality recorded with the transmittance probe on the tongue was higher (*P* = 0.0066) and the perfusion index was lower (*P* < 0.0001) compared with measurements with the reflectance probe placed on the tail. In group DEX, signal quality of the measurements from the tongue was higher compared with measurements from the tail (*P* = 0.0009); Tables 6, 7).

**Table 6:** Analysis of the signal quality measured by a transmittance pulse oximeter probe placed on the tongue or by a reflectance pulse oximeter probe placed on the tail or tibia in anesthetized dogs that had or had not received dexmedetomidine.

	***n***	**Signal quality m (min-max)**	**Signal quality m (min-max)**	***P*-value**
**Tongue vs. tail**		**Tongue**	**Tail**	
BUT	132	10/10 (4–10/10)	10/10 (3–10/10)	**0.0066**
DEX	100	10/10 (3–10/10)	9/10 (3–10/10)	**0.0009**
**Tongue vs. tibia**		**Tongue**	**Tibia**	
BUT	92	10/10 (9–10/10)	10/10 (3–10/10)	**<0.0001**
DEX	60	10/10 (3–10/10)	5/10 (3–10/10)	**<0.0001**
**Tail vs. tibia**		**Tail**	**Tibia**	
BUT	85	10/10 (3–10/10)	10/10 (3–10/10)	**<0.0001**
DEX	49	10/10 (3–10/10)	5/10 (3–10/10)	**0.0002**

*Values were only included if measurements for both positions were available and analyzed as paired values*.

*n, number of measurements; tongue, transmittance pulse oximeter probe placed on the tongue; tail, reflectance pulse oximeter probe placed at the ventral tail base; tibia, transmittance pulse oximeter probe placed on the medial aspect of the proximal tibia; BUT, premedication butorphanol 0.2 mg/kg IV; DEX, premedication dexmedetomidine 5 μg/ g + butorphanol 0.2 mg/kg IV; m, median. Bold values mean significant differences*.

**Table 7:** Analysis of the perfusion index measured by a transmittance pulse oximeter probe placed on the tongue or by a reflectance pulse oximeter probe placed on the tail and tibia in anesthetized dogs that had or had not received dexmedetomidine.

	***n***	**Perfusion index m (min-max)**	**Perfusion index m (min-max)**	***P*-value**
**Tongue vs. tail**		**Tongue**	**Tail**	
BUT	132	3/10 (1–10/10)	5/10 (1–10/10)	**<0.0001**
DEX	100	2/10 (1–10/10)	3/10 (1–10/10)	0.2904
**Tongue vs. tibia**		**Tongue**	**Tibia**	
BUT	92	3/10 (1–10/10)	3/10 (1–9/10)	0.0744
DEX	60	2/10 (1–9/10)	2/10 (1–10/10)	0.2134
**Tail vs. tibia**		**Tail**	**Tibia**	
BUT	85	5/10 (1–10/10)	3/10 (1–9/10)	**<0.0001**
DEX	49	3/10 (1–8/10)	2/10 (1–10/10)	0.3677

*Values were only included if measurements for both positions were available and analyzed as paired values*.

*n, number of measurements; tongue, transmittance pulse oximeter probe placed on the tongue; tail, reflectance pulse oximeter probe placed on the ventral tail base; tibia, transmittance pulse oximeter probe placed on the medial aspect of the proximal tibia; BUT, premedication butorphanol 0.2 mg/kg IV; DEX, premedication dexmedetomidine 5 μg/kg + butorphanol 0.2 mg/kg IV; m, median. Bold values mean significant differences*.

## Discussion

The aim of this study was to evaluate the performance of reflectance pulse oximetry with the probe attached to the base of the tail or the tibia in anesthetized dogs, some of which had received dexmedetomidine (vasoconstrictor agent). The results were compared with those generated by conventional transmittance pulse oximetry with the probe placed on the tongue. Measurements using the tibia and tail as probe sites had a higher failure rate when compared with the tongue. The failure rate was higher when the tibia was used compared with the tail.

Pulse oximeters require adequate pulsation and well-perfused tissues to generate proper readings (9, 10). When tissues are poorly perfused, pulse oximeters underestimate the oxygen saturation and may lose the signal (11, 12). New-generation pulse oximeters, similar to the one used in this study, are equipped with advanced technologies that improve their performance in poorly perfused tissue (13). Reflectance pulse oximeter probes can record signals that are up to 10 times weaker than the minimum required by transmittance probes. Therefore, hypoperfusion as well as patient movement and ambient light can interfere with readings generated by reflectance pulse oximetry (14, 15). Anatomical structures with well-perfused tissue and underlying bone for reflection of the red and infrared light can be challenging to find for placement of a reflectance probe. Fixation of a reflectance probe in some positions is difficult, and the tail base has been suggested as an alternative site for probe placement (16). The conical shape of the ventral tail base means that slight movement of the tail can disconnect the reflectance probe from the skin and interrupt the signal. The medial aspect of the tibia presents an even more challenging probe site because of the concave shape of the tuberosity. These factors may explain the higher failure rate when the tibia and the tail base were used as probe placement sites. The higher failure rate seen when using the tibia rather than the tail base may have been because the probe can be attached more securely to the tail base, reducing artifacts and ambient light. In addition, the tissue between the bone and the probe may be better perfused in the tail compared with the medial aspect of the proximal tibia. Other possible probe positions include the palmar metatarsus and metacarpus, but these need to be evaluated in future studies.

Vasoactive drugs, such as dexmedetomidine, affect peripheral perfusion and thus the accuracy of pulse oximeters (17). This was confirmed in the present study because the failure rate of all probe sites was higher when dogs were premedicated with dexmedetomidine. In contrast to these findings, other studies found that reflectance pulse oximeter probes had a similar or better performance than transmittance probes when used in poorly perfused tissue (8, 18, 19). A possible reason for this discrepancy is that hypoperfusion was obtained experimentally using hypothermia or vasopressor drugs in the human studies. Furthermore, the oximeter probe was attached to the forehead in these studies and it is possible that the changes in local perfusion in response to vasoconstrictive drugs differ between the forehead and the sites used in the present study. We used dexmedetomidine, which causes moderate local hypoperfusion attributable to vasoconstriction.

Compared with values obtained from the tongue, the SpO_2_ was higher in measurements from the tail in both premedication groups. The gold standard for the assessment of oxygenation is arterial blood gas analysis, which was not done in this study because we did not apply for an animal experimentation permit. The accuracy of two new-generation pulse oximeters (Masimo Rad-5 and Edan H100N) was evaluated in dogs, and the results were compared with arterial blood gas analysis as the gold standard (20). The Masimo Rad-5 pulse oximeter equipped with a transmittance probe underestimated arterial oxygen saturation (SaO_2_) by 3–4%. In anesthetized sheep, a similar underestimation was seen with the same pulse oximeter that was used in the present study (21). The accuracy of SpO_2_ compared with SaO_2_ using a reflectance probe was also evaluated in anesthetized horses and revealed a deviation of −1.3 to 3.1% (22). Based on these studies, the reflectance probe seems to provide an even more accurate SpO_2_ value than the transmittance probe, and the differences in SpO_2_ recorded by the pulse oximeter probes in the present study are likely not clinically relevant.

An SpO_2_ of < 90% was observed in some dogs. However, all dogs had received 100% oxygen, had an adequate minute ventilation, and did not have underlying pulmonary disease. In addition, the multiparameter monitor displayed an SpO_2_ of 95% or more. Therefore, we assume that the dogs were not hypoxic but rather that the pulse oximeter readings were inaccurate. These findings were more common in group DEX; as an α-2-agonist, dexmedetomidine induces vasoconstriction, resulting in reduced tissue perfusion, bradycardia, and respiratory sinus arrhythmia (23, 24).

In both premedication groups, signal quality and perfusion index were significantly higher when the tail was used for probe placement compared with the tibia. This suggests that the tail is the more reliable probe site for attachment of the reflectance probe than the tibia.

A major limitation of this study was the lack of arterial blood gas analysis as a gold standard of oxygenation. Further studies should be undertaken to provide information about the accuracy of the reflectance pulse oximeter probe relative to arterial blood gas analysis.

Local perfusion, and therefore signal quality, may have been affected by the depth of anesthesia, which was at the discretion of the anesthesiologist. Further studies should also investigate the reliability of the reflectance probe in non-anesthetized patients.

Depending on the type of premedication, 72–88% of the readings had acceptable signal quality. Therefore, the ventral base of the tail can be considered an adequate probe position for reflectance pulse oximetry in dogs. This is particularly useful in situations in which the nature of the procedure renders the tongue inaccessible for probe placement. Pulse rate and SpO_2_ values measured by the pulse oximeter should only be interpreted when signal quality is adequate.

## Data Availability Statement

The raw data supporting the conclusions of this article will be made available by the authors, without undue reservation.

## Ethics Statement

The animal study was reviewed and approved by Ethics committee of the Centre of Clinical Veterinary Medicine, LMU Munich (number 16-04-10-13). Written informed consent was obtained from the owners for the participation of their animals in this study.

## Author Contributions

JN: study design, data collection, and preparation of the manuscript. YZ: statistical analysis. KH: study design and critical revision of the manuscript. RD: study design, statistical analysis, and critical revision of the manuscript. All authors contributed to the article and approved the submitted version.

## Conflict of Interest

The authors declare that the research was conducted in the absence of any commercial or financial relationships that could be construed as a potential conflict of interest.

## Footnotes

^1^Dexmedetomidine (Dexdomitor 0.1 mg/ml, Orion Corporation, Espoo, Finland).

^2^Butorphanol (Alvegesic, 10 mg/ml, CP Pharma, Burgdorf, Germany).

^3^Propofol (Narcofol, 10 mg/ml, CP Pharma, Burgdorf, Germany).

^4^Rüschelit^®^ Super Safety Clear (Teleflex Medical GmbH, Fellbach, Germany).

^5^Sevoflurane (SevoFlo Abbott Laboratories Ltd, Berkshire, UK).

^6^Nihon Kohden Monitor BSM-2301 (Nihon Kohden, Tokyo, Japan).

^7^Rad5^®^ (Masimo Corporation, Irvine, USA).

^8^LNCS TC-I clip sensor (Masimo Corporation, Irvine, USA).

^9^LNCS TF-I reflecting sensor (Masimo Corporation, Irvine, USA).

^10^Prism 5 for Windows (Graph Pad Software, Inc. San Diego, USA).
